# Recent advances in radiation therapy of pancreatic cancer

**DOI:** 10.12688/f1000research.16272.1

**Published:** 2018-12-13

**Authors:** Bhanu Prasad Venkatesulu, Cheng-En Hsieh, Keith L Sanders, Sunil Krishnan

**Affiliations:** 1 Department of Experimental Radiation Oncology, University of Texas MD Anderson Cancer Center, Houston, TX, USA; 2 Department of Radiation Oncology, University of Texas MD Anderson Cancer Center, Houston, TX, USA; 3 The University of Texas MD Anderson Cancer Center-UT Health Graduate School of Biomedical Sciences, Houston, TX, USA; 4 Department of Radiation Oncology, Chang Gung Memorial Hospital, Linkou and Chang Gung University, Taoyuan, Taiwan

**Keywords:** Dose escalation, immunotherapy, neoadjuvant therapy, spleen sparing, radiotherapy

## Abstract

Pancreatic cancer has a dismal prognosis with an overall survival outcome of just 5% at five years. However, paralleling our improved understanding of the biology of pancreatic cancer, treatment paradigms have also continued to evolve with newer advances in surgical techniques, chemotherapeutic agents, radiation therapy (RT) techniques, and immunotherapy paradigms. RT dose, modality, fraction size, and sequencing are being evaluated actively, and the interplay between RT and immune effects has opened up newer avenues of research. In this review, we will emphasize recent advances in RT for pancreatic cancer, focusing on preoperative chemoradiation, RT dose escalation, sparing of the spleen to reduce lymphopenia, and combination of RT with immunotherapy.

## Introduction

Pancreatic cancer is the second most common gastrointestinal cancer in the United States, where there was an estimated incidence of 53,070 cases in 2016 ^1^. Survival outcomes are dismal with a reported five-year overall survival (OS) rate of less than 5% ^2^. Radiation therapy (RT) is an integral component of the arsenal against pancreatic cancer and is frequently used in the neoadjuvant, adjuvant, and palliative settings. In an ever-changing landscape of treatment options for pancreatic cancer, some patterns are emerging that bear upon the utilization of RT in this disease. A recent autopsy study concluded that—independent of initial clinical stage, histological features, and treatment course—30% of patients die with locally destructive pancreatic cancer whereas 70% die with distant metastatic disease ^3^. Patients in this sizeable subset tend to lack inactivation of their *DPC4* gene and may benefit considerably from intensified treatment with a local treatment modality. In agreement with this, post-pancreatectomy patterns-of-recurrence studies have shown that patients most commonly (about 50–60%) recur systemically but that a large subset recur locally (20–25%) or both locally and distantly (15–20%). Again, local treatment modalities may play a significant role in minimizing local recurrence, and a greater understanding of biological predictors of varied patterns of recurrence should help elucidate potential ways to reduce the risk of recurrence ^4– 6^. Although these studies suggest that there is a role for local treatment intensification, there is little agreement on whether RT improves survival in patients by addressing this local recurrence risk, when this should be administered, how it is best administered, and what other principles dictate efficacy of treatment ^7– 9^. We address these gaps in knowledge in the next few sections and highlight advances in our understanding of the role of radiotherapy in pancreatic cancer.

## Defining a borderline resectable pancreatic cancer subset

Historically, localized non-metastatic pancreatic cancers were categorized as locally advanced or resectable on the basis of the likelihood of major vascular involvement on radiographic studies that dictated whether or not a margin-negative R0 resection was feasible. Radiographic assessment sheds light on involvement of the retroperitoneal margin—superior mesenteric artery (SMA) and superior mesenteric vein (SMV)—as well as that of the celiac artery and confluence of the SMV and the portal vein (PV). More recently, a new category of borderline resectable pancreatic cancer (BRPC) has been created wherein there is some degree of involvement of vascular structures that could compromise achievement of an R0 resection. A number of groups have tried to define criteria for resectability ^10, 11^. The consensus includes anatomic criteria such as feasibility of reconstruction of SMV-PV confluence, less than 180° involvement of the SMA and celiac axis, and common hepatic artery origin from celiac axis that can be reconstructed. Some groups also include in this category patients with uncertainty regarding metastatic stage usually arising from indeterminate distant lesions on radiographic studies and poor performance status that would preclude immediate surgery ^11^.

The creation of a category of BRPC dovetails with the recognition that the technically challenging nature of resection along the retroperitoneal margin results in patients frequently undergoing an incomplete (margin-positive) resection ^12– 18^. In many studies, resection margin status—in addition to tumor size, stage, and grade—is considered an important predictor of early recurrence and inferior survival rates ^15, 16, 18– 22^. This poor survival rate ^20, 23, 24^ closely mimics that of patients with locally advanced pancreatic cancer (LAPC) ^25^. Furthermore, postoperative chemoradiation therapy (CRT) does not overcome the unfavorable prognosis conferred by a margin-positive resection ^12, 14^. Therefore, leaving behind a positive margin at surgery is essentially akin to not having undergone surgery at all since the survival outcomes are no better than those of LAPC patients who did not undergo surgical resection in the first place ^12, 14, 20, 23– 25^. Notably, during the recovery time from this surgery, patients are unable to receive chemotherapy or RT, further hampering early and effective disease control locally and distantly. Recognizing the detrimental effect of margin-positive resections and creating a BRPC category allow better selection of patients for surgical resection. Importantly, they also offer the opportunity to deliver some neoadjuvant therapy to patients with BRPC to make them potentially resectable and to reduce the likelihood that this resection will be a margin-positive R1 resection.

## Preoperative chemoradiation

Preoperative therapy has been explored by a number of groups in both potentially resectable pancreatic cancer and BRPC. While the optimal treatment approaches for all categories of non-metastatic pancreatic cancer remain controversial, for BRPC, the most compelling argument for neoadjuvant therapy is to convert them to potentially resectable tumors by sterilizing the retroperitoneal margin. For potentially resectable pancreatic cancers, a number of arguments can be made for neoadjuvant therapy. In pancreatic cancers, as in other cancers where preoperative therapy is used routinely (rectal and esophageal) ^26, 27^, preoperative treatment allows (a) better tissue penetration of drug and oxygen, resulting in greater response to chemotherapy and radiotherapy, respectively; (b) greater chances of an R0 resection (since even potentially resectable pancreatic cancers that go to surgery upfront often encounter positive margins and perineural invasion); (c) better outcomes even if the resection ends up being R1 ^28^; (d) lower risk of anastomotic leaks at the pancreaticojejunostomy site because of firmer post-radiation pancreatic tissue that sutures go through ^29– 31^; and (e) the ability to select patients who pass the stress test of neoadjuvant treatment satisfactorily and do not develop interval metastases or newly diagnosed, previously occult metastatic disease (both good performance status and true non-metastatic localized disease serving as clinical indicators of a favorable biology). By relegating the most challenging treatment (that is, surgery) to the end of the treatment course, this approach also ensures a high probability of patients receiving all three components of treatment (surgery, chemotherapy, and radiotherapy) rather than suffer from delays in or abandonment of treatment (20–30% of patients are unable to receive planned postoperative therapy) because of prolonged recovery times from surgery or new metastatic disease manifesting itself during the recovery periods ^32^.

A number of phase II studies have evaluated the role of CRT in the neoadjuvant setting for potentially resectable pancreatic cancer. A meta-analysis ^33^ noted that neoadjuvant therapy for initially resectable tumors resulted in a 4% complete radiographic or pathologic response rate, 31% partial radiographic or pathologic response rate, 74% resectability, and a median OS of 23.3 months, which is similar to that of patients who undergo surgery upfront. In the first prospective randomized phase II trial, the German investigators compared surgery followed by six cycles of gemcitabine to neoadjuvant gemcitabine, cisplatin, and RT (50.4 Gy to the regional nodes and 55.8 Gy to the tumor) followed by surgery and six cycles of adjuvant gemcitabine chemotherapy. The study was terminated early after enrollment of only a quarter of planned patients because of slow accrual but noted a median OS of 17.4 months with neoadjuvant CRT compared with 14.4 months with adjuvant chemotherapy arm ( *P* = 0.96) with comparable R0 and pN0 resections in both arms ^34^. When patients with BRPC are also included, the potential benefit of neoadjuvant therapy seems to be more apparent, as was hinted at in the meta-analysis above. The meta-analysis noted that, for initially unresectable BRPC and LAPC, neoadjuvant therapy resulted in 5% radiographic or pathologic complete response rate, 30% partial response rate, 33% resectability, and a median OS of 20.5 months, which is comparable to that of initially resectable patients.

Early results of the multi-center randomized phase III PREOPANC-1 study presented recently at the American Society of Clinical Oncology 2018 annual meeting compared preoperative CRT with adjuvant chemotherapy in 246 patients, split nearly evenly between BRPC and potentially resectable patients ^35^. The preoperative therapy arm received a cycle of gemcitabine followed by gemcitabine and RT (36 Gy in 15 fractions) and another cycle of gemcitabine before surgery and four cycles of postoperative gemcitabine, whereas the adjuvant chemotherapy arm received six cycles of gemcitabine. Preoperative treatment resulted in improved median OS (17.1 versus 13.5 months, *P* = 0.74) and greater time to recurrence (9.9 versus 7.9 months, *P* = 0.023) and R0 resection rate (63% versus 31%, *P* <0.001) without any difference in toxicity profiles. Notably, among patients who underwent surgery, the median OS was significantly better in the neoadjuvant arm than in the adjuvant arm (29.9 versus 16.8 months, *P* <0.001). Although these results are promising and suggest that patients with BRPC should receive neoadjuvant therapy followed by re-evaluation for resectability, the ideal approach would be to treat patients on protocols to establish the value of neoadjuvant therapy more objectively. One such study that is accruing currently is the randomized phase II Alliance for Clinical Oncology trial (A021501) where patients with BRPC receive FOLFIRINOX (oxaliplatin, irinotecan, leucovorin, and infusional 5-fluorouracil) alone or FOLFIRINOX followed by stereotactic body RT (SBRT) of 33–40 Gy in five fractions before surgery ^36^.

## Radiation dose escalation in pancreatic cancer

Radiation dose escalation has been studied the most in LAPC where treatment outcomes are poorer than for resectable and borderline resectable patients and the likelihood of conversion to resectability is low. The randomized phase III LAP-07 trial showed that CRT of 54 Gy with gemcitabine after 4 months of gemcitabine-based chemotherapy did not provide an OS benefit compared with 6 months of chemotherapy alone in LAPC but CRT was associated with reduced local progression (32% versus 46%, *P* = 0.03). The findings of this study suggest that standard-dose RT produces good local control but treatment intensification may be warranted to improve OS ^37^. Treatment intensification could be achieved via more potent chemotherapy such as FOLFIRINOX or gemcitabine-Nab-paclitaxel or via radiation dose escalation. As systemic therapy becomes more effective, the local failure may become a cause for greater concern if it exceeds the nearly 30% of patients who were noted to die with locally destructive pancreatic cancer in the autopsy series described previously.

**Figure 1.**
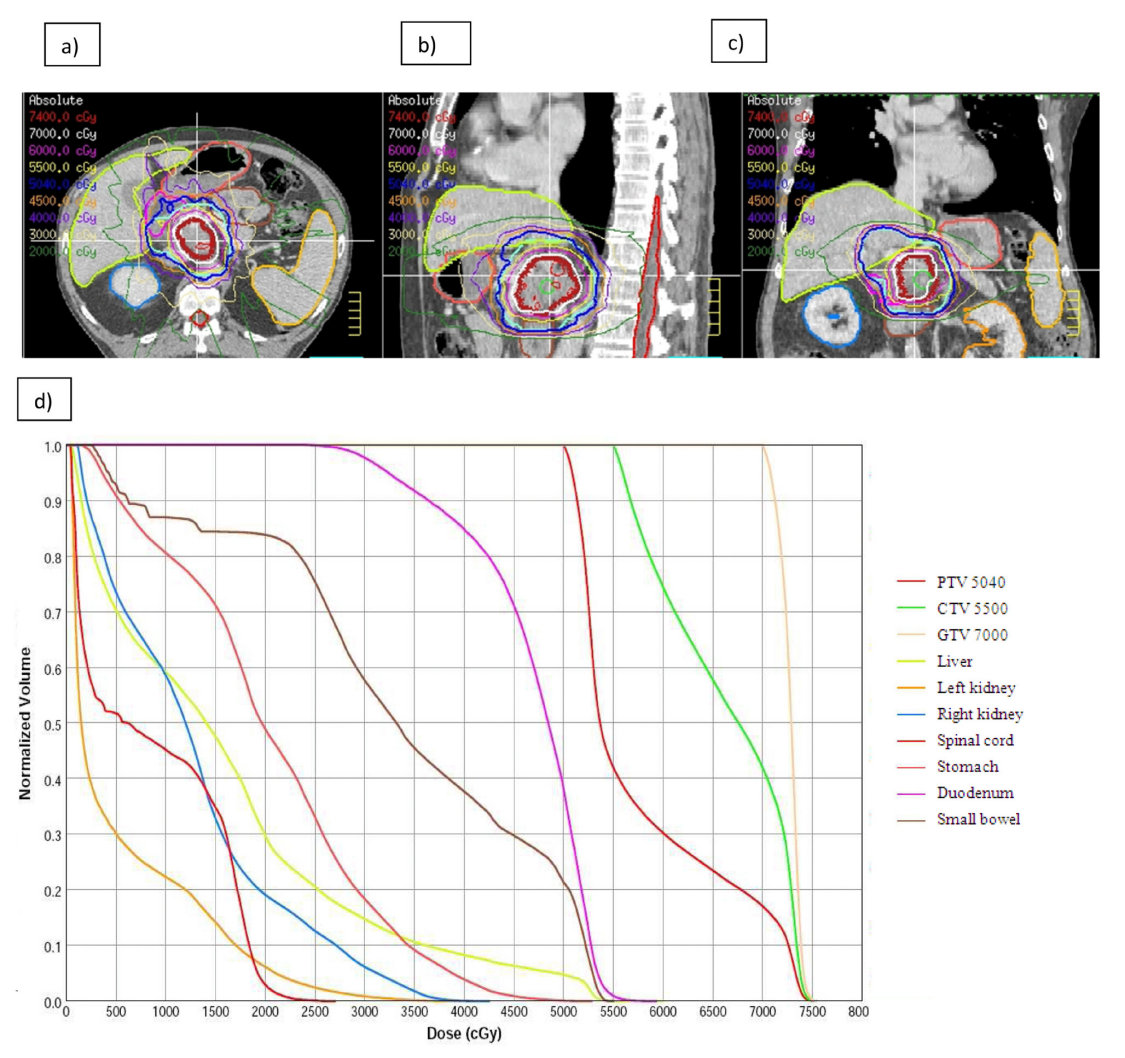
Representative images and treatment plan for dose-escalated radiation therapy for pancreatic cancer. Axial ( **a**), sagittal ( **b**), and coronal ( **c**) view of a patient who received 50.4 Gy in 28 fractions to the planning target volume with simultaneous integrated boost of 55 Gy to the clinical target volume and 70 Gy boost to the gross tumor volume. ( **d**) Dose volume histogram of the targets and organs at risk. This figure was produced by SK, ( **a**), ( **b**) and ( **c**) uses original images taken in SK’s clinic for this publication.

A viable option for improving local control is to escalate the dose of RT to the tumor while respecting normal tissue dose constraints. This can be accomplished via standard courses of fractionated RT of about five weeks with incremental increases in dose-per-fraction to the planning target volume with the option of an integrated boost to the gross tumor volume typically achieved with intensity-modulated RT (IMRT) or via SBRT, where higher doses of radiation are administered to small tumor volumes with precise image guidance and motion management. Figure 1 highlights a patient who had dose escalation of 70 Gy to the gross tumor volume through IMRT. In both instances, the close proximity of gastrointestinal mucosa (stomach, duodenum, and jejunum) precluded excessive dose escalation. In a phase III randomized French study, gemcitabine alone was compared with 5-fluorouracil, cisplatin, and 60 Gy of RT to a large pancreatic field followed by gemcitabine ^38^. The chemotherapy-alone arm fared better with a median OS of 13 months compared with 8.6 months ( *P* = 0.03) but was also significantly less toxic. The unusually low OS on the CRT arm indicated that radiation dose escalation is potentially deleterious if performed without image guidance and if combined with overly intensive chemotherapy. On the other hand, Chung *et al*. reported that dose escalation of more than 61 Gy leads to better one-year OS and progression-free survival (PFS) ^39^. Krishnan *et al*. reported that patients who received a biologically equivalent dose (BED) greater than 70 Gy had superior OS (17.8 versus 15 months, *P* = 0.03) as well as better local PFS (10.2 versus 6.2 months, *P* = 0.05) compared with those receiving BED of less than 70 Gy ^40^. It is noteworthy that this trial reported outcomes from the start of RT and not from the date of diagnosis and yet the high BED cohort had a remarkable three-year OS of 31%. Admittedly, this was possible in only a subset of patients whose tumors were at least 1 cm away from bowel mucosa and required daily image guidance, motion tracking and control, and maintenance of an empty stomach for 3 hours before treatment in the majority of patients. Most patients did not require fiducial placement since daily volumetric imaging was available on a CT-on-rails. With similar attention to organ motion and image guidance, the other approach that is convenient and easily incorporated into the overall management plan of patients is SBRT in which a high BED can be administered in a short duration. Again, respecting bowel mucosal constraints is critical since this is an organ with serial functional subunits (like the spinal cord) and excessive dose to even a small volume could result in significant functional compromise and toxicity. With gentler fractionation of five fractions, a moderate dose of 33 Gy has now been safely administered to a large number of patients. A multi-institutional phase II study demonstrated that gemcitabine with SBRT of 33 Gy in five fractions over the course of one week is safe and technically feasible in LAPC ^41^. As noted above, the Alliance for Clinical Oncology Trial A021501 seeks to gently increase the radiation dose up to 40 Gy in five fractions in patients with BRPC, bringing it closer to ablative doses of radiation used in the treatment of liver tumors ^36^. By intensifying the systemic therapy as well as the local therapy, this trial will address both causes of failure in patients.

An alluring option that is worth considering is that of being less stringent with defining who proceeds to surgery after neoadjuvant CRT. For instance, among the first large reports of BRPC treatment with preoperative CRT, only 41% of 160 patients who completed preoperative therapy and restaging advanced to pancreatectomy, 94% of which were margin-negative pancreatectomies ^42^. One could argue that the very low rates of margin positivity suggest that resectability criteria are too stringent. More recently, it was noted that neoadjuvant FOLFIRINOX did not convert locally advanced and borderline resectable patients to resectable by radiographic criteria in a majority of patients; even so, when taken to surgery, the R0 resection rate was 92% ^43^. This suggests that traditional radiological criteria for resectability may overestimate the likelihood of margin positivity and that adoption of less stringent criteria may increase the number of patients going to surgery without a large increase in margin positivity. Extended to its logical conclusion, this conjecture would result in a three-pronged fork in the road after completion of CRT. The first prong (say, the good) would be those patients with clear traditional radiological criteria for resectability who would go to surgery. The second (say, the bad) would be those patients with traditional radiological criteria that would not warrant surgery but there are no adverse factors such as worsening performance status, questionable new metastases, unexplained tumor marker increase, or complete encasement of celiac axis or SMA. They merely have involvement of the SMA or celiac axis that precludes resectability by traditional radiological criteria. These patients would undergo reimaging in a couple of months and, if all criteria remain unchanged, would undergo surgery with an implicit acceptance of a higher likelihood of R1 resections. The third (say, the ugly) would be patients with encasement of vessels, worsening performance status, questionable new metastases, and rising tumor markers. These patients would not go to surgery. While treatment paradigms are slowly shifting in this direction for BRPC, such a trend is lacking in LAPC. Treatment of patients on protocols may help define the value of such an approach more objectively.

## Spleen as a dose-limiting organ in pancreatic cancer radiotherapy

A preponderance of evidence suggests that lymphopenia, especially that following RT, confers a poor prognosis in the treatment of a variety of cancers ^44^. This was also observed in pancreatic cancer in a study from Johns Hopkins University where post-treatment lymphopenia correlated with increased mortality in resectable pancreatic cancer and LAPC. RT toxicity to circulating lymphocytes was postulated as the likely cause of lymphopenia that was independent of chemotherapy usage. Mortality was reported to be due to tumor progression rather than lymphopenia-related opportunistic infections ^45, 46^. A report from the MD Anderson Cancer Center also demonstrated that post-CRT lymphopenia occurred in roughly a third of all patients with LAPC and was strongly associated with poor survival outcomes. Importantly, however, the dose to the spleen (mean dose exceeding 9 Gy and V15 exceeding 20%) was an independent predictor of post-CRT lymphopenia, suggesting that the detrimental effect of lymphopenia is potentially minimized by sparing the spleen ^47^. The spleen routinely receives unintentional radiation dose while irradiating pancreatic cancers not only because it lies in close proximity to the pancreas but also because it is not commonly viewed as a dose-limiting organ while developing treatment plans. However, it is a rich reservoir of T and B lymphocytes that slowly traverse through the sinusoidal architecture of flow channels within it, allowing ample time for collateral radiation injury during each fraction of treatment. Conceivably, this depletion of resident or slow-moving lymphocytes in the spleen could also reduce the levels of CD8 ^+^ cytotoxic T lymphocytes and CD4 ^+^ helper T cells available for tumor infiltration. In turn, this could adversely impact OS outcomes as noted in surgical series where less tumor infiltration with CD8 ^+^ T cells is associated with poorer OS ^48, 49^ and reduce the effectiveness of future potential immunotherapies that rely on the presence of a pool of healthy lymphocytes for activation and tumor homing ^50^. Of note, tumor size, field size, irradiated volume (encompassed by the 50% isodose line), and treated volume (encompassed by the 95% isodose line) did not independently predict the likelihood of developing lymphopenia, suggesting that depletion of circulating lymphocytes may not be a dominant mechanism of developing lymphopenia in patients with pancreatic cancer.

The correlation between splenic dose and lymphopenia needs to be independently validated in other series. However, since it is unlikely that prospective studies will establish the clinical benefit of spleen-sparing RT for pancreatic cancer, treatment plans could be readily triaged on the basis of their ability to adequately spare the spleen. In patients most vulnerable to developing lymphopenia or having poor outcomes from lymphopenia such as patients with baseline lymphopenia prior to initiating CRT, patients who are candidates for immunotherapy, and patients being considered for RT dose escalation protocols, one could consider using spleen-sparing beam angles for three-dimensional conformal RT, IMRT, increased dose rates for beam delivery, charged particle therapy, and SBRT ^51, 52^. More refined normal tissue complication probability modeling may also serve as a predictive tool to spatially correlate splenic dose volume histograms with lymphopenia.

Alternatively, strategies could be developed to replenish or protect lymphocytes (or both) from damage mediated by RT. Preclinical models have demonstrated that interleukin-7 (IL-7) and IL-15 administration to irradiated mice resulted in greater tumor regression which was associated with an increase in CD8 ^+^ cytotoxic T cell counts without an increase in immunosuppressive regulatory T cell counts ^53– 55^. A phase I clinical trial is exploring IL-7 administration in high-grade glioma patients who develop lymphopenia after completion of CRT ^56^. IL-15 is being evaluated as monotherapy in metastatic melanoma and renal cell carcinoma, albeit not for lymphopenia ^57^. It would be worthwhile to evaluate the role of IL-15 as a rescue agent for lymphopenia following RT. Lymphocyte reconstitution is frequently used as a rescue strategy following myeloablative chemotherapy and RT in transplant protocols and adoptive T-cell therapy ^58^. In one study, autologous lymphocytes harvested before temozolomide–RT for high-grade gliomas were reinfused after completion of treatment. However, the return of lymphocyte counts following radiation-induced depletion was no different between reinfused patients and matched controls ^59^.

## Immunotherapy with radiation in pancreatic cancer

Consistent with the notion that lymphocytes play a crucial role in determining treatment outcomes of these patients, pancreatic cancer has also been the focus of immuno-oncology studies aimed at arousing the immune system to combat cancer progression and metastasis. However, pancreatic cancer poses a unique challenge to immunotherapy because of the presence of a thick fibrous capsule with intense desmoplastic stroma that counteracts the entry of immune cells. In addition, the pancreatic tumor milieu has a preponderance of regulatory T cells, myeloid-derived suppressor cells, and M2 macrophages that contribute to the immunosuppressive tumor microenvironment ^60^. Pancreatic cancer tends to produce IL-10, transforming growth factor-beta (TGF-β), and increased expression of programmed death-ligand 1 (PD-L1) that prevents activation of tumor antigen-specific T cells.

Nevertheless, accumulating evidence of a synergy between immunotherapy and radiotherapy has resulted in enormous growth of this area as a promising and exciting avenue of research. Herein, RT is seen as serving as an *in situ* vaccine via release of autoantigens and radiation-induced neoantigens that are displayed to antigen-presenting cells that then cross-present tumor antigen to prime T cells to mount a cytotoxic tumor-specific immune response. RT triggers the elaboration of a damage-associated molecular pattern (DAMP) response wherein cells display hallmarks of the “eat me” phenotype that leads to phagocytosis by antigen-presenting cells. In turn, these antigen presenting cells cross-present tumor antigens to T cells which enable immune-mediated tumor killing ^61^. But RT can also be immunosuppressive by upregulating the expression of death receptors and immune checkpoint proteins that drive co-inhibitory pathways to evade immune eradication. Also, RT can induce lymphocyte apoptosis via secretion of galectin-1 by tumors and lead to secretion of TGF-β into the tumor microenvironment, thereby hindering the ability to mount an effective cytotoxic T-cell response to tumor antigens ^62– 64^. Careful tuning of this balance between immunostimulatory and immunosuppressive effects of radiation can lead to a dominance of immune stimulation and immune-mediated tumor eradication. Preclinical studies suggest that an ablative dose of RT has the potential for potent T-cell priming in draining lymph nodes, facilitation of increased antigen presentation, activation of immune response–related genes, radiation-induced DAMP molecules, and increased release of inflammatory cytokines.

SBRT may have a unique role in synergizing with immunotherapy because the high dose induces a dominant *in situ* vaccination effect and the small number of fractions leads to less depletion of primed and activated cytotoxic T cells returning to the tumor ^65^. Currently, over 30 clinical trials are evaluating immunotherapy in pancreatic cancer. Algenpantucel-L is an alpha-1, 3-galactosyl transferase–expressing allogeneic pancreatic tumor cell vaccine that is supposed to cause rapid activation of antibody-dependent cell-mediated cytotoxicity (toward pancreatic cancer cells). GVAX is a cancer vaccine that has been genetically modified to produce granulocyte-macrophage colony-stimulating factor (GM-CSF) that induces a robust T-cell response. Both of these agents are being evaluated with SBRT ^66, 67^. Losartan, via inhibition of the renin-angiotensin system, downregulates TGF-β activity, thereby facilitating intratumoral penetration of drugs by stromal and vascular remodeling. Studies are evaluating the combination of losartan and PD-L1 blockade with SBRT. Other studies are exploring the combination of immune checkpoint inhibitors—antibodies to cytotoxic T lymphocyte–associated protein 4 (CTLA-4) and PD-L1—with SBRT and chemotherapy to find the optimal combination that generates durable treatment responses and improved OS ^68– 71^. Although immunotherapy holds promise in pancreatic cancer, pragmatic clinical trials with optimal sequencing of immunotherapy and RT are needed ^72^. Finding innovative ways to enhance the permeability of the pancreatic cancer stromal matrix for efficient drug delivery may hold the key to enhancing the effect of immunotherapy, especially because metastases are as stroma-rich as the primary tumor ^73^; radiation may play a role in magnifying the role of immunotherapeutic agents through judicious elaboration of its *in situ* vaccine effect. Table 1 summarizes the currently active clinical trials that combine radiotherapy with immunotherapy in pancreatic cancer.

**Table 1.** Summary of the currently active clinical trials that are ongoing that combine radiation with immunotherapy in pancreatic cancer.

ClinicalTrials.gov Identifier	Phase	Title	Intervention	Radiation detail	Disease stage
NCT02648282	2	Study With CY, Pembrolizumab, GVAX, and SBRT in Patients With Locally Advanced Pancreatic Cancer	Cyclophosphamide GVAX Pembrolizumab	6.6 Gy per day over the course of 5 days	Locally advanced pancreatic cancer
NCT03104439	2	Nivolumab and Ipilimumab and Radiation Therapy in MSS and MSI High Colorectal and Pancreatic Cancer	Nivolumab Ipilimumab	8 Gy per day over the course of 3 days	Oligometastatic pancreatic cancer
NCT03161379	2	Phase 2 GVAX Pancreas Vaccine (With CY) in Combination With Nivolumab and SBRT for Patients With Borderline Resectable Pancreatic Cancer	Cyclophosphamide Nivolumab GVAX	6.6 Gy per day over the course of 5 days	Borderline Resectable Pancreatic Adenocarcinoma
NCT02305186	1/2	Safety and Immunological Effect of Pembrolizumab in Resectable or Borderline Resectable Pancreatic Cancer (UVA-PC- PD101)	Pembrolizumab Capecitabine	50.4 Gy in 28 fractions over the course of 28 days	Resectable or Borderline Resectable Pancreatic Cancer
NCT03563248	2	Losartan and Nivolumab in Combination With FOLFIRINOX and SBRT in Localized Pancreatic Cancer	5-Fluorouracil Oxaliplatin Irinotecan Leucovorin Losartan Nivolumab	SBRT	Borderline/potentially resectable or locally advanced.

CY, cyclophosphamide; GVAX, granulocyte-macrophage colony-stimulating factor (GM-CSF) gene-transfected tumor cell vaccine; MSI, microsatellite instable; MSS, microsatellite stable; SBRT, stereotactic body radiotherapy.

## Conclusions

Exciting new clinical research suggests that the outcomes in pancreatic cancer can be improved by a multi-pronged approach. Careful stratification of patients into distinct categories, adoption of neoadjuvant therapy for resectable and borderline resectable categories, judicious focal dose escalation with image guidance and motion management, avoidance of splenic irradiation to reduce lymphopenia, and potentially the combination of RT with immunotherapeutic agents may all have a role in the optimal management of pancreatic cancer with RT in the future. For now, these approaches address critical challenges faced while considering RT during the treatment of pancreatic cancer and only continued evaluation of such strategies on clinical trials will address persisting gaps in knowledge.
